# Supplementary material for: Characterization of Traditional Chinese Sesame Oil by Using Headspace Solid-Phase Microextraction/Gas Chromatography–Mass Spectrometry, Electronic Nose, Sensory Evaluation, and RapidOxy
Source: Foods. 2022 Nov 8;11(22):3555. doi: 10.3390/foods11223555 (PMC9689288; doi:10.3390/foods11223555)
Supplement: Supplementary file 1 [file foods-11-03555-s001.zip › foods-1972891-supplementary.pdf]

Table S1. Identification and quantification of volatile compounds in XMXY1-5, RSO and CSO samples.

NO	Formula	Compound[a]	CAS	RI1$_{cal}$[b]	RI1$_{ref}$[c]	RI2$_{cal}$[d]	RI2$_{ref}$[e]	Relative concentration(ug/g)						
								XMXY1	XMXY2	XMXY3	XMXY4	XMXY5	RSO	CSO
1	CH4S	Methanethiol	74-93-1	<600	<600	674	675	0.00±0.00	0.62±0.16	1.58±0.61	0.64±0.25	1.02±0.15	0.43±0.25	0.00±0.00
2	C3H6O2	Methyl acetate	79-20-9	<600	<600	817	813	0.00±0.00	0.00±0.00	3.63±0.28	0.00±0.00	4.05±0.42	0.00±0.00	0.00±0.00
3	C4H8O	2-Butanone	78-93-3	<600	<600	895	894	0.00±0.00	0.00±0.00	1.54±0.1	0.00±0.00	1.32±0.52	0.00±0.00	0.00±0.00
4	C6H8O	2,5-Dimethyl-furan	625-86-5	703	696	931	946	0.00±0.00	0.00±0.00	0.55±0.1	0.34±0.17	0.00±0.00	0.00±0.00	0.00±0.00
5	C5H10O	Pentanal	110-62-3	695	699	969	971	0.00±0.00	0.00±0.00	0.00±0.00	0.00±0.00	0.00±0.00	0.28±0.11	0.00±0.00
6	C2H6S2	Dimethyl disulfide	624-92-0	739	736	1047	1070	0.39±0.17	0.00±0.00	2.49±0.11	0.93±0.2	1.75±0.31	0.00±0.00	0.00±0.00
7	C6H12O	Hexanal	66-25-1	799	780	1061	1073	0.00±0.00	0.00±0.00	0.00±0.00	0.00±0.00	0.00±0.00	0.00±0.00	2.03±0.72
8	C5H7N	N-methyl-pyrrole	96-54-8	nd	nd	1119	1127	0.00±0.00	0.00±0.00	0.6±0.16	0.00±0.00	0.00±0.00	0.00±0.00	0.00±0.00
9	C5H5N	Pyridine	110-86-1	736	749	1178	1173	0.81±0.05	0.00±0.00	0.00±0.00	1.41±0.05	0.00±0.00	0.45±0.05	0.00±0.00
10	C4H4N2	Pyrazine	290-37-9	728	734	1196	1204	1.39±0.06	2.22±0.64	5.05±1.26	2.35±0.29	2.94±0.2	0.00±0.00	0.00±0.00
11	C3H3NS	Thiazole	288-47-1	742	745	1231	1238	0.33±0.01	0.00±0.00	0.00±0.00	0.00±0.00	0.00±0.00	0.00±0.00	0.00±0.00
12	C8H8	Styrene	100-42-5	890	895	nd	nd	0.00±0.00	0.00±0.00	0.00±0.00	0.00±0.00	0.00±0.00	0.71±0.14	0.00±0.00
13	C5H6N2	Methyl-pyrazine	109-08-0	820	829	1250	1259	36.83±1.51	38.34±13.17	61.86±15.18	41.3±6.5	43.07±3.58	28.51±11.4	0.00±0.00
14	C4H5NS	4-Dethyl-thiazole	693-95-8	813	802	1262	1265	1.94±0.17	2.18±0.55	4.13±0.52	2.82±0.55	3.52±0.62	0.00±0.00	0.00±0.00
15	C5H7NS	2,4-Dimethyl-thiazole	541-58-2	880	883	1265	1268	0.00±0.00	0.00±0.00	0.00±0.00	0.00±0.00	0.00±0.00	1.93±1.07	0.00±0.00
16	C6H8N2	2,5-Dimethyl-pyrazine	123-32-0	910	914	1309	1311	15.82±0.98	13.44±4.17	27.2±4.76	0.00±0.00	0.00±0.00	18.26±1.88	0.00±0.00
17	C6H8N2	2,3-Dimethyl-pyrazine	5910-89-4	914	915	1335	1337	1.87±0.05	3.9±1.28	3.34±1.05	2.2±0.93	1.74±0.33	0.98±0.72	0.00±0.00
18	C2H6S3	Dimethyl trisulfide	3658-80-8	968	973	1344	1354	1.78±0.16	0.00±0.00	0.00±0.00	0.00±0.00	0.00±0.00	0.00±0.00	0.00±0.00
19	C7H10N2	2-Ethyl-6-methyl-pyrazine	13925-03-6	995	994	1366	1367	6.72±0.5	5.87±1.03	14.62±2	7.86±1.06	6.34±0.15	3.21±1.1	0.00±0.00
20	C7H10N2	2-Ethyl-5-methyl-pyrazine	13360-64-0	1000	997	1373	1376	7.07±0.76	4.84±1.4	9.41±2.52	5.74±1.1	4.76±0.28	4.68±1.33	0.00±0.00
21	C7H10N2	2-Ethyl-3-methyl-pyrazine	15707-23-0	999	999	1388	1391	3.45±0.36	2.43±0.72	5.75±1.09	3.3±1.03	2.6±0.15	1.65±0.2	0.00±0.00
22	C7H10N2	Trimethyl-pyrazine	14667-55-1	999	999	1392	1394	6.69±0.69	6.44±3.42	12.77±2.57	6.74±1.97	5.62±0.34	6.68±0.62	0.00±0.00
23	C6H6N2	Ethenyl-pyrazine	4177-16-6	928	926	1422	1429	0.94±0.29	1.19±0.66	1.96±0.63	0.00±0.00	0.00±0.00	0.00±0.00	0.00±0.00
24	C2H4O2	Acetic acid	64-19-7	<600	<600	1425	1427	7.16±0.45	7.47±1.02	6.1±1.49	0.00±0.00	22.79±3.83	30.75±10.64	0.72±0.13
25	C8H12N2	3-Ethyl-2,5-dimethyl-pyrazine	13360-65-1	1073	1069	1430	1430	11.7±1.36	10.18±2.03	19.57±4.16	10.07±4.78	10.35±1.23	8.77±3.48	0.00±0.00
26	C5H4O2	Furfural	98-01-1	827	nd	nd	nd	15.74±1.09	17.53±7	0.00±0.00	11.83±6.02	19.85±7.22	0.00±0.00	0.00±0.00
27	C6H6O2	1-(2-furanyl)-Ethanone	1192-62-7	906	910	1488	1488	0.00±0.00	0.00±0.00	0.00±0.00	5.15±1.33	0.00±0.00	0.00±0.00	0.00±0.00
28	C7H6O	Benzaldehyde	100-52-7	960	960	1506	1502	2.5±0.28	3.1±2.21	0.00±0.00	4.7±0.59	0.00±0.00	1.41±0.26	0.00±0.00
29	C7H8O3	Furfuryl acetate	623-17-6	989	991	1512	1529	0.00±0.00	0.00±0.00	3.08±0.17	3.31±0.78	2.92±0.61	0.00±0.00	0.00±0.00
30	C3H6O2	Propanoic acid	79-09-4	672	nd	1515	nd	0.00±0.00	0.00±0.00	2.66±0.39	0.00±0.00	1.45±0.31	0.00±0.00	0.06±0.02
31	C6H6O3	Methyl 2-furoate	611-13-2	970	978	1557	1553	0.88±0.01	1.07±0.56	0.00±0.00	1.91±0.19	1.76±0.36	0.00±0.00	0.00±0.00
32	C6H6O2	5-Methyl-2-furancarboxaldehyde	620-02-0	957	957	1560	1597	16.91±1.04	18.07±5.62	4.93±1.79	14.05±3.17	24.54±4.25	3.03±0.93	0.00±0.00
33	C6H7NO	3-Methoxy-pyridine	7295-76-3	nd	nd	1579	1579	0.73±0.06	0.7±0.28	0.47±0.13	1.88±0.17	1.62±0.27	0.00±0.00	0.00±0.00

34	C7H7NO	2-Acetylpyridine	1122-62-9	nd	nd	1586	1590	0.79±0.03	0.56±0.44	1.85±0.55	1.12±0.1	0.52±0.22	0.00±0.00	0.00±0.00
35	C6H6N2O	Acetylpyrazine	22047-25-2	1019	1021	1615	1618	12.14±1.06	0.00±0.00	6.51±2.16	9.05±0.78	0.00±0.00	0.00±0.00	0.00±0.00
36	C5H5NOS	2-Acetylthiazole	24295-03-2	nd	nd	1627	1634	2.16±0.18	0.00±0.00	1.68±0.48	0.00±0.00	0.00±0.00	0.00±0.00	0.00±0.00
37	C8H8O	Acetophenone	98-86-2	1064	1065	nd	nd	0.00±0.00	0.00±0.00	5.62±1.33	0.00±0.00	0.00±0.00	0.00±0.00	0.00±0.00
38	C5H6O2	2-Furanmethanol	98-00-0	848	852	1644	1651	13.36±0.67	16.02±2.92	18.61±4.58	16.18±4.46	19.19±4.32	0.00±0.00	0.00±0.00
39	C7H8N2O	1-(6-methyl-2-pyrazinyl)-1-Ethanone	22047-26-3	1111	1095	1687	1679	0.00±0.00	5.87±2.11	3.71±2.23	6.9±1.51	10.16±1.62	4.23±1.88	0.00±0.00
40	C6H8O2	5-Methyl-2-furanmethanol	3857-25-8	947	953	nd	nd	0.00±0.00	0.00±0.00	3.58±1.33	0.00±0.00	0.00±0.00	0.00±0.00	0.00±0.00
41	C6H6OS	3-Acetyl-thiophene	1468-83-3	nd	nd	1757	1771	0.00±0.00	0.00±0.00	0.46±0.18	0.00±0.00	0.00±0.00	0.00±0.00	0.00±0.00
42	C6H8OS2	2-[(methyldithio)methyl]-Furan	57500-00-2	1213	1226	1775	1806	0.86±0.23	0.00±0.00	1.25±0.27	0.78±0.13	0.00±0.00	0.00±0.00	0.00±0.00
43	C6H8O2	2-Hydroxy-3-methyl-2-cyclopenten-1-one	80-71-7	1023	1028	1824	1824	0.00±0.00	0.00±0.00	0.69±0.23	0.00±0.00	0.8±0.46	0.00±0.00	0.00±0.00
44	C5H10O2	Pentanoic acid	109-52-4	910	911	1822	nd	0.00±0.00	0.00±0.00	0.61±0.27	0.00±0.00	0.00±0.00	0.00±0.00	0.39±0.03
45	C6H12O2	Hexanoic acid	142-62-1	nd	nd	1827	nd	0.00±0.00	0.00±0.00	0.00±0.00	0.00±0.00	0.00±0.00	0.00±0.00	2.99±0.31
46	C7H8O2	2-Methoxy-phenol	90-05-1	1084	nd	1844	nd	14.84±12.65	28.36±8.51	85.49±25.26	54.78±8.78	45.22±6.38	5.56±1.35	0.00±0.00
47	C7H8O	Benzyl alcohol	100-51-6	nd	nd	1864	1864	0.00±0.00	0.13±0.03	1.27±0.75	0.66±0.14	0.00±0.00	0.00±0.00	0.00±0.00
48	C8H10O	Phenylethyl alcohol	60-12-8	nd	nd	1899	nd	0.00±0.00	0.00±0.00	0.87±0.22	0.73±0.36	0.00±0.00	0.00±0.00	0.00±0.00
49	C4H8O2	Butanoic acid	107-92-6	824	826	1910	nd	0.00±0.00	0.00±0.00	0.00±0.00	0.00±0.00	0.00±0.00	0.00±0.00	0.14±0.01
50	C6H7NO	2-Acetyl-1H-pyrrole	1072-83-9	1061	1063	1580	1950	5.36±0.53	5.71±1.74	9.64±3.41	10.43±2.24	10.26±1.57	2.5±0.72	0.00±0.00
51	C6H6O	Phenol	108-95-2	nd	nd	1992	1992	1.21±0.11	1.25±0.41	0.00±0.00	1.83±0.27	1.72±0.28	0.00±0.00	0.00±0.00
52	C5H5NO	1H-pyrrole-2-carboxaldehyde	1003-29-8	1012	1015	nd	nd	13.54±1.36	13.13±4.67	5.35±2.61	15.17±4.3	22.92±4.06	6.81±2.64	0.00±0.00
53	C9H12O2	4-Ethyl-2-methoxy-phenol	2785-89-9	1276	1279	2017	2008	0.00±0.00	0.00±0.00	13.06±4.09	2.42±0.67	2.38±0.61	0.00±0.00	0.00±0.00
54	C8H16O	3-Methyl-4-heptanone	15726-15-5	nd	nd	2021	nd	0.00±0.00	0.00±0.00	0.00±0.00	0.00±0.00	0.00±0.00	4.07±0.74	0.00±0.00
55	C6H7NO2	Methyl pyrrole-2-carboxylate	1193-62-0	nd	nd	2042	2058	0.39±0.04	0.38±0.06	0.00±0.00	0.00±0.00	0.00±0.00	0.00±0.00	0.00±0.00
56	C8H16O2	Octanoic acid	124-07-2	nd	nd	2044	2041	0.00±0.00	0.00±0.00	0.27±0.15	0.00±0.00	0.00±0.00	0.00±0.00	0.00±0.00
57	C4H7NO	2-Pyrrolidinone	616-45-5	nd	nd	2066	2029	0.00±0.00	0.00±0.00	0.00±0.00	0.00±0.00	0.00±0.00	9.66±1.2	0.00±0.00
58	C7H8O	P-cresol	106-44-5	826	1059	1129	2091	0.00±0.00	0.00±0.00	0.34±0.32	0.00±0.00	0.00±0.00	0.00±0.00	0.00±0.00
59	C6H7NO	1-Methyl-1H-pyrrole-2-carboxaldehyde	1192-58-1	1001	1010	2108	1620	3.22±0.29	3.11±1	2.24±0.99	3.12±0.91	6.12±1.24	2.05±0.76	0.00±0.00
60	C9H18O2	Nonanoic acid	112-05-0	1268	1272	2148	2144	1.03±0.53	0.00±0.00	0.00±0.00	0.00±0.00	0.00±0.00	0.00±0.00	0.00±0.00
61	C9H10O2	2-Methoxy-4-vinylphenol	7786-61-0	1309	1309	2191	2194	4.75±0.51	5.22±1.95	4.08±1.64	4.55±1.65	23.78±5.54	4.3±1.72	0.00±0.00
62	C8H6O3	Piperonal	120-57-0	1329	1329	2236	2244	0.00±0.00	0.00±0.00	0.26±0.17	0.00±0.00	0.00±0.00	0.00±0.00	0.00±0.00
63	C8H10O3	2,6-Dimethoxy-phenol	91-10-1	1348	nd	2270	nd	0.00±0.00	0.00±0.00	2.31±0.8	0.00±0.00	0.00±0.00	0.00±0.00	0.00±0.00
64	C6H9NOS	4-Methyl-5-thiazoleethanol	137-00-8	1271	1271	2305	2309	0.00±0.00	0.00±0.00	0.00±0.00	0.00±0.00	0.00±0.00	0.91±0.33	0.00±0.00
65	C3H8O3	Glycerin	56-81-5	nd	nd	2329	2322	0.00±0.00	1.69±0.5	22.65±4.7	0.57±0.09	0.00±0.00	0.00±0.00	0.00±0.00
66	C10H12O2	2-Methoxy-4-(1-propenyl)-phenol	97-54-1	1452	1448	2152	2350	0.00±0.00	0.00±0.00	0.14±0.06	0.00±0.00	0.00±0.00	0.00±0.00	0.00±0.00
67	C8H7N	Indole	120-72-9	1292	1295	2441	2441	0.00±0.00	0.00±0.00	0.00±0.00	0.00±0.00	2.28±0.86	0.00±0.00	0.00±0.00
68	C9H10O3	Apocynin	498-02-2	nd	nd	2681	2676	0.00±0.00	0.00±0.00	0.81±0.27	0.00±0.00	0.00±0.00	0.00±0.00	0.00±0.00
69	C7H6O3	1,3-Benzodioxol-5-ol	533-31-3	1300	1312	2687	nd	4.24±0.47	8.06±2.98	5.56±2.38	19.19±4.84	15.51±2.89	4.24±1.98	0.00±0.00

Compound[a]: Volatile compounds detected in the three different processed sesame oil samples.

RI1$_{cal}$[b]: retention index calculated of compounds on WAX capillary column.

RI2$_{cal}$[d]: retention index calculated of compounds on DB-5MS capillary column.

RI1$_{ref}$[c], RI2$_{cal}$[e]: retention indices obtained from NIST standard reference database (https://webbook.nist.gov/chemistry/) (accessed on 29 September 2022).

XMXY1-5: Xiao Mo Xiang You 1-5; RSO: roasted sesame oil; CSO: cold pressed sesame oil.

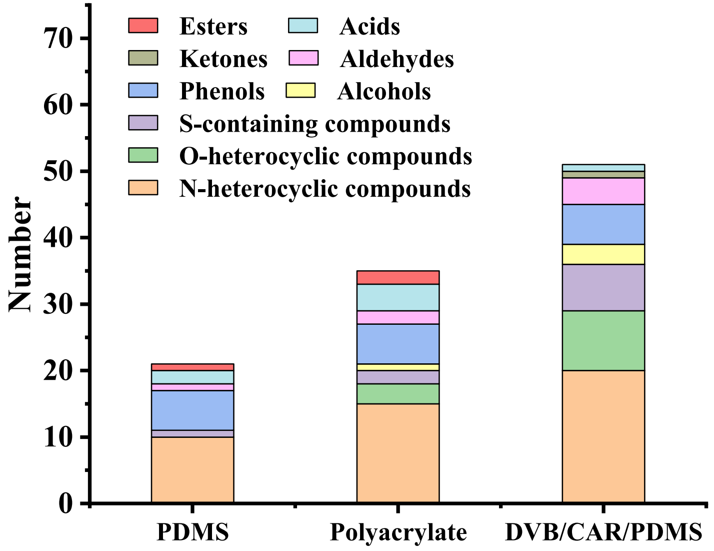

Figure S1. Comparison of three kinds of fibers for volatile extraction efficiency.

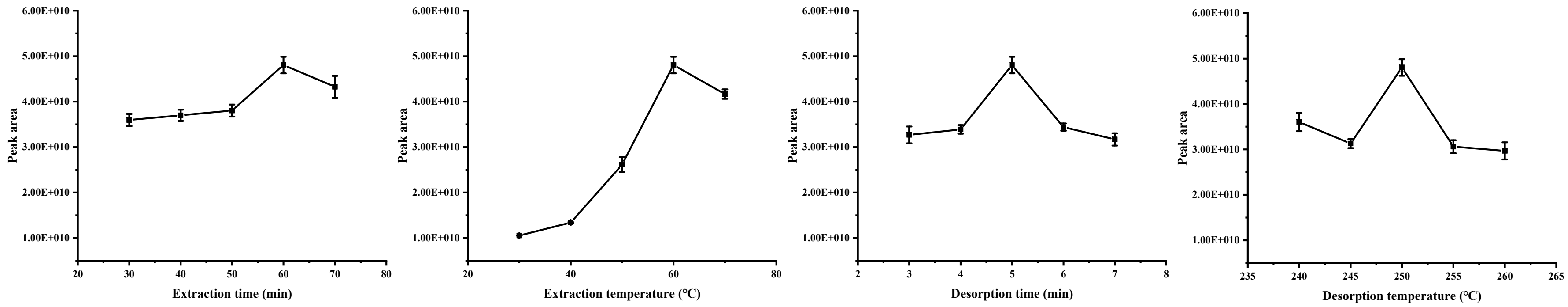

(**A**) (**B**) (**C**) (**D**)

Figure S2. Comparison of HS-SMPE conditions: (A) extraction time, (B) extraction temperature, (C) desorption time (D) desorption temperature.
